# Mycobacterial acyl carrier protein suppresses TFEB activation and upregulates miR-155 to inhibit host defense

**DOI:** 10.3389/fimmu.2022.946929

**Published:** 2022-09-28

**Authors:** Seungwha Paik, Kyeong Tae Kim, In Soo Kim, Young Jae Kim, Hyeon Ji Kim, Seunga Choi, Hwa-Jung Kim, Eun-Kyeong Jo

**Affiliations:** ^1^ Department of Microbiology, Chungnam National University School of Medicine, Daejeon, South Korea; ^2^ Department of Medical Science, Chungnam National University School of Medicine, Daejeon, South Korea; ^3^ Infection Control Convergence Research Center, Chungnam National University School of Medicine, Daejeon, South Korea

**Keywords:** *Mycobacterium tuberculosis*, transcription factor EB, phagosome-lysosome fusion, microRNA-155-5p, acyl carrier protein, bone marrow-derived macrophages

## Abstract

Mycobacterial acyl carrier protein (AcpM; Rv2244), a key protein involved in *Mycobacterium tuberculosis* (Mtb) mycolic acid production, has been shown to suppress host cell death during mycobacterial infection. This study reports that mycobacterial AcpM works as an effector to subvert host defense and promote bacterial growth by increasing microRNA (miRNA)-155-5p expression. In murine bone marrow-derived macrophages (BMDMs), AcpM protein prevented transcription factor EB (TFEB) from translocating to the nucleus in BMDMs, which likely inhibited transcriptional activation of several autophagy and lysosomal genes. Although AcpM did not suppress autophagic flux in BMDMs, AcpM reduced Mtb and LAMP1 co-localization indicating that AcpM inhibits phagolysosomal fusion during Mtb infection. Mechanistically, AcpM boosted the Akt-mTOR pathway in BMDMs by upregulating miRNA-155-5p, a SHIP1-targeting miRNA. When miRNA-155-5p expression was inhibited in BMDMs, AcpM-induced increased intracellular survival of Mtb was suppressed. In addition, AcpM overexpression significantly reduced mycobacterial clearance in C3HeB/FeJ mice infected with recombinant *M. smegmatis* strains. Collectively, our findings point to AcpM as a novel mycobacterial effector to regulate antimicrobial host defense and a potential new therapeutic target for Mtb infection.

## Introduction

Tuberculosis (TB) is a worldwide infectious disease that has claimed many lives, and the fight against TB still faces many challenges. According to the World Health Organization’s global TB report 2020, TB caused an estimated 10 million new cases and 1.5 million deaths in 2020, making it the second most deadly infectious disease caused by a single pathogen after COVID-19. *Mycobacterium tuberculosis* (Mtb), the bacteria that causes tuberculosis, has a variety of defense mechanisms to evade the host’s innate immune system, including autophagy, apoptosis, and inflammation (1). Mtb can also survive as a latent infection for a long time in alveolar macrophages, making it resistant to anti-TB drugs and difficult to eradicate (2). To control Mtb, it’s crucial to understand the dynamics of the host-pathogen interaction. To date, several mycobacterial factors, such as SapM (3), ESAT-6/CFP-10 (4), nuoG (5), Eis (6), LprG (7), PE_PGRS47 (8), SecA2 (9, 10), LprE (11), PknG (12), and phthiocerol dimycocerosates (PDIM) (13), are known to influence how Mtb suppresses host defenses through modulating various innate immune strategies against Mtb in host immune cells. Nonetheless, new mycobacterial components that alter the host’s innate immune response must be discovered to better understand the molecular mechanisms underlying mycobacterial pathogenesis and develop new therapeutic targets.

Mtb requires a unique acyl carrier protein (AcpM), the second most glycosylated protein involved in mycolic acid biosynthesis (14). Mycolic acids, which protect Mtb from the host environment while also eluting virulence, are one of the most important components of the mycobacterial cell wall (15). AcpM interacts with PptT, which transfers 4′-phosphopantetheine (Ppt) from coenzyme A (CoA) to AcpM in Mtb for mycolic acid synthesis (16). According to a recent study, a small compound called “8918” inhibited PptT action by binding to the Ppt pocket in the active site, resulting in selective antimicrobial activity comparable to rifampin (17). These findings raise concerns about the intrinsic properties of the AcpM and how they affect Mtb virulence. Although AcpM is essential for Mtb growth by producing lipid-rich cell walls, little is known about its immunological properties in host-pathogen interactions.

This study investigated the mechanisms by which the AcpM protein prevents nuclear translocation of transcription factor EB (TFEB) and phagosomal maturation in host macrophages. AcpM appeared to inhibit autophagy in bone marrow-derived macrophages (BMDMs) by lowering the LC3 I to II ratio; however, it did not affect autophagic flux in BMDMs. Rather than this, AcpM markedly reduced nuclear translocation of TFEB and several autophagy-related genes including lysosomal-associated membrane protein 1 *(Lamp1)*, which was regulated by TFEB, in macrophages. Moreover, AcpM activated the protein kinase B (Akt) pathway, which is associated with Mtb survival in host cells, by inducing miR-155, which targets SH2-domain-containing inositol 5-phosphatase 1 (SHIP1) (18). AcpM prevented Mtb from fusing with lysosomes in BMDMs, thus increasing Mtb intracellular survival (ICS). Finally, in the lung lysates of recombinant *M. smegmatis*-infected mice, AcpM overexpression increased Mtb colony-forming unit (CFU) levels while decreasing several autophagy and lysosomal genes.

Taken together, these findings help us to explore the relationship between the host immune response and mycobacterial infection in terms of Mtb AcpM, revealing its potential as a target for novel tuberculosis therapies.

## Materials and methods

### Animals and ethics statement

Female C57BL/6 and BALB/c mice were purchased from Samtako Bio (Gyeonggi-do, Korea) at 6–7 weeks of age, and C3HeB/FeJ mice were obtained from the Jackson Laboratory (Bar Harbor, ME, USA). Mice were maintained under specific pathogen-free conditions. All animal experimental methods and procedures were performed following the relevant ethical guidelines and regulations approved by the Institutional Research and Ethics Committee at Chungnam National University, School of Medicine (202009A-CNU-155; Daejeon, Korea) and the guidelines of the Korean Food and Drug Administration.

### Cell culture

Bone marrow cells were isolated from C57BL/6 mice (6-8 weeks old) and cultured in Dulbecco’s modified Eagle’s medium (DMEM; Lonza, Walkersville, USA) containing 10% fetal bovine serum (FBS; Gibco, NY, USA) and antibiotics (Lonza). Differentiating for 4–5 days in the presence of 25 μg/ml of recombinant mouse macrophage colony-stimulating factor (M-CSF) (R&D Systems) in a 37°C humidified atmosphere containing 5% CO_2_ produced primary BMDMs. Approximately 4 x 10^5^ cells/well in the 24-well cell culture plate (SPL Life Science Co., Gyeonggi-do, Korea) or 2 x 10^5^ cells/well in the 48-well cell culture plate (Corning, NY, USA) were used for the entire *in-vitro* analysis.

### Preparation of recombinant AcpM protein and anti-AcpM antibody

Recombinant AcpM protein was prepared according to the previous study (19). Briefly, mycobacterial *acpM* was amplified from genomic DNA of Mtb H37Rv ATCC 27294 using the forward (5’-CATATGCCTGTCACTCAGGAAGAAATC-3’) and reverse primers (5’-AAGCTTCTTGGACTCGGCCTCAAGCCT-3’), and the PCR product was inserted into the pET-22b (+) vector (Novagen, Madison, WI, USA). The recombinant plasmids were transformed into *E. coli* BL21 cells by heat-shocking for 1 min at 42 °C. Cell disruption was used to obtain the overexpressed AcpM protein, which was then purified using NI-NTA resin. The purified recombinant protein was dialyzed and incubated with polymyxin B-agarose (Sigma Chemical Co.) to remove residual endotoxin. The purified endotoxin-free AcpM was filter sterilized and kept frozen at -80°C until use. To collect anti-AcpM antibodies, BALB/c mice were injected three times intraperitoneally with purified AcpM (25 μg per mouse) emulsified in incomplete Freund’s adjuvant. One week after the final immunization, serum was collected and stored frozen until use with proper dilution.

### Construction of recombinant *M. smegmatis* strains

Mycobacterial *acpM* was amplified from genomic DNA of Mtb H37Rv ATCC 27294 using the forward (*Nde*I site, 5’-CATATGCCTGTCACTCAGGAAGAAATC-3’) and reverse primers (*Hin*dIII site, 5’-AAGCTTCTTGGACTCGGCCTCAAGCCT-3’) as in the previous study (19). Then, amplified *acpM* was inserted into the pVV16 vector to create pVV16_AcpM. The pVV16 (vector only) and pVV16_AcpM plasmids were electroporated into suspensions of *M. smegmatis* mc^2^155 competent cells at 2.5 kV, 1,000 Ω, and 25 μF using a Gene Pulser (Bio-Rad, San Diego, CA, USA) to construct Ms_Vec and Ms_AcpM, respectively. Western blot image of AcpM expression in Ms_Vec and Ms_AcpM using anti-AcpM antibody was presented in **Supplementary Figure S1**.

### Western blot analysis

BMDMs cultured in 24-well cell culture plates were lysed in 150 μl of radioimmunoprecipitation assay (RIPA) buffer (LPS solution, CBR002) added with protease and phosphatase inhibitor cocktail (Roche, Mannheim, Germany). The whole mouse lung was homogenized in 1 ml of PBS containing 0.05% Tween 80 (PBST) and then half of the homogenates were centrifuged and lysed in 500 μl of RIPA buffer containing protease and phosphatase inhibitor cocktail. The cell lysates were mixed with Protein 5X Sample Buffer (ELPIS BIOTECH, EBA-1052) and boiled for 10 min. Prepared protein extracts were separated by SDS-polyacrylamide gel electrophoresis (PAGE) and then transferred to polyvinylidene difluoride (PVDF; Millipore, Burlington, MA, USA) membranes. The membranes were then blocked using 1X blocking solution (Biofact) for 1 h at room temperature (RT) and then incubated overnight with primary antibodies at 4 °C. After washing with tris-buffered saline supplemented with 0.1% Tween 20 (TBST), the membranes were incubated with the secondary antibodies for 1 h at RT. Immunoblotting was performed using an enhanced chemiluminescence reagent (Millipore, WBKL S0500) and a UVitec Alliance mini-chemiluminescence device (UVitec, Rugby, UK). The densitometric values were calculated using ImageJ software and data were normalized to loading controls shown in the figures. Bafilomycin A1 (B1793) was purchased from Sigma-Aldrich (St. Louis, MO, USA) The primary and secondary antibodies used were as follows: Anti-p62 (1:1000 diluted; P0067) and anti-LC3 (1:1000 diluted; L8918) antibodies were purchased from Sigma-Aldrich. anti-LAMP1 (1:1000 diluted; sc-20011) was purchased from Santa Cruz Biotechnology (Dallas, TX, USA), Anti-β-actin (1:2000 diluted; 5125s), anti-phospho-mTOR (1:1000 diluted; 2971s), anti-mTOR (1:1000 diluted; 2983s), anti-phospho-Akt (1:1000 diluted; 4060s), anti-Akt (1:1000 diluted; 9272s), anti-TFEB (1:1000 diluted; 4240s), anti-ATG5 (1:1000 diluted; 12994s), anit-SHIP1 (1:1000 diluted; 2728s), anti-FOXO3a (1:1000 diluted; 12829s), anti-mouse IgG (1:5000 diluted; 7076s), and anti-rabbit IgG (1:5000 diluted; 7074s) antibodies were purchased from Cell Signaling Technology (Danvers, MA, USA).

### Bacterial strains and culture

Mtb H37Rv was kindly provided by Dr. R. L. Friedman (University of Arizona, Tucson, AZ, USA). Mtb was grown at 37 °C with shaking in Middlebrook 7H9 broth (Difco, Paris, France) supplemented with 0.5% glycerol, 0.05% Tween-80 (Sigma-Aldrich), and oleic albumin dextrose catalase (OADC; BD Biosciences). Mtb-expressing enhanced red fluorescent protein (Mtb-ERFP) and recombinant *M. smegmatis* strains were grown in Middlebrook 7H9 medium supplemented with OADC and 50 μg/ml kanamycin (Sigma-Aldrich). Bacterial strains were then harvested by centrifugation at 3000 rates per min for 30 min and the pellets were resuspended in ice-cold phosphate-buffered saline (PBS). All mycobacterial suspensions were aliquoted and stored at −80 °C until just before use. For all experiments, mid-log-phase bacteria (O.D = 0.6) were used. The number of CFUs of the inoculum was verified by serially diluting and plating on Middlebrook 7H10 agar (Difco).

### Immunofluorescence analysis

BMDMs were cultured on coverslips in 24-well cell culture plates. After the appropriate infection or treatment, cells were washed twice with PBS, fixed with 4% paraformaldehyde for 15 min, and permeabilized with 0.25% Triton X-100 (Sigma-Aldrich) for 10 min. Cells were incubated with anti-TFEB antibody (1:400 diluted; Bethyl Laboratories, A303-673A) or anti-LAMP1 Ab (1:400 diluted; Santa Cruz Biotechnology, SC-19992) overnight at 4°C. Cells were washed with PBS to remove excess primary antibodies and then incubated with secondary anti-rabbit or anti-rat IgG-Alexa Fluor 488 Ab (1:400 diluted; Invitrogen, A11008 or A11006) for 1 h at RT. Nuclei were stained using Fluoromount-G™, with DAPI mounting medium (Thermo Fisher Scientific, 00-4959-52). Immunofluorescence images were acquired using a confocal laser-scanning microscope (Zeiss, LSM-900). Quantification of TFEB-nuclear translocation was performed by manual calculation and the degree of colocalization between Mtb-ERFP and LAMP-1 was analyzed using the JACoP plugin of the ImageJ software.

### Total RNA extraction and sequencing

Total RNA from BMDMs was isolated using QIAzol lysis reagent (Qiagen, Hilden, Germany) and miRNeasy Mini Kits (Qiagen) according to the manufacturer’s instructions. RNA quality was assessed by Agilent 2100 bioanalyzer using the RNA 6000 Pico Chip (Agilent Technologies, CA, USA), and quantification was performed using a NanoDrop 2000 Spectrophotometer system (Thermo Fisher Scientific, MA, USA). For messenger RNA-sequencing (mRNA-seq), the library was constructed using QuantSeq 3’ mRNA-Seq Library Prep Kit (Lexogen, Wien, Austria) according to the manufacturer’s instructions. In brief, each sample was prepared with 500 ng of total RNA, an oligo-dT primer with an Illumina-compatible sequence at its 5’ end was hybridized with the RNA, and reverse transcription was performed. After degradation of the RNA template, second-strand synthesis was initiated by a random primer with an Illumina-compatible linker sequence at its 5’ end. The double-stranded library was purified using magnetic beads to remove all reaction components and amplified to add the complete adapter sequences required for cluster generation. The finished library was purified from PCR components, and then high-throughput sequencing was performed as single-end 75 sequencings using NextSeq 500 (Illumina, CA, USA). For micro RNA-sequencing (miRNA-seq), the construction of the library was performed using the NEBNext Multiplex Small RNA Library Prep kit (New England BioLabs, MA, USA) according to the manufacturer’s instructions. Briefly, for library construction, total RNA from each sample was used 1 µg to ligate the adaptors, and then cDNA was synthesized using reverse-transcriptase with adaptor-specific primers. PCR was performed for library amplification, and libraries were cleaned up using QIAquick PCR Purification Kit (Qiagen) and AMPure XP beads (Beckman Coulter, CA, USA). The Agilent 2100 Bioanalyzer instrument assessed the yield and size distribution of the small RNA libraries for the High-sensitivity DNA Assay (Agilent Technologies). The NextSeq500 system produced High-throughput sequences to single-end 75 sequencings (Illumina).

All raw reads received the quality check using BBduk, a tool in the BBMap package (https://sourceforge.net/projects/bbmap), to remove low-quality bases (< Q20). The remaining reads from QuantSeq 3’ mRNA-Seq and miRNA-seq were mapped to the mouse mm10 genome reference and mature miRNA sequences of the miRBase database (20) using Bowtie2 software (21), respectively. Read counts of genes were calculated with Bedtools (22) and the raw counts were transformed into counts per million (CPM) for exclusion of very lowly expressed genes using edgeR (version 3.36.0) (23). Genes with one or more log2-CPM in at least two samples were kept for further analysis. Next, normalization factors were calculated with the trimmed mean of M-values (TMM) method using the calcNormFactors function in edgeR. For Z-score normalization, the TMM-adjusted log CPM counts were calculated, and Gaussian normalization was performed. To identify differentially expressed genes (DEGs), gene expression levels were statistically tested between groups using the glmFit and glmLRT functions embedded in the edgeR package. Benjamini and Hochberg’s false discovery rate (FDR) method was used to correct for multiple testing. Genes with the fold change over two and the significance (adjusted p-value) below 0.01 were considered DEGs. The binding site between miRNA and the 3’ untranslated region (UTR) of target mRNA was predicted by miRWalk 3.0 at http://mirwalk.umm.uni-heidelberg.de/ (last accessed February 2022).

### Quantitative real-time PCR

For mRNA expression analysis, total RNA from BMDMs cultured in 48-well cell culture plates or mouse lung tissue homogenates was extracted using TRIzol reagent (Invitrogen; 15596026) according to the manufacturer’s instructions, followed by RNA quantitation and assessment using QIAxpert (Qiagen). Complement DNA from total RNA was synthesized using the reverse transcription master premix (ELPIS Biotech; EBT-1515c) as manufacturer’s instruction. Two-step quantitative real-time PCR (qRT-PCR) was carried out using cDNA, primers, and Rotor-Gene SYBR Green PCR Kit (Qiagen, 204074). Reactions were run on a Rotor-Gene Q 2plex system (Qiagen, 9001620). The samples were amplified for 40 cycles as follows: 95°C for 5 s and 60°C for 10 s. Data were expressed as relative fold changes using the 2-ΔΔ threshold cycle (Ct) method with *β-actin* (BMDMs) or *Gapdh* (lung tissue homogenates) as an internal control gene. The primer sequences used are shown in **Supplementary Table 1**.

For miRNA expression analysis, total RNA from BMDMs cultured in 48-well cell culture plates was isolated using QIAzol lysis reagent (Qiagen, 79306) and miRNeasy Mini Kits (Qiagen, 217004) according to the manufacturer’s instructions. Next, cDNA from total RNA was synthesized using miScript II RT Kits (Qiagen, 218161) by the manufacturer’s instructions. Three-step qRT-PCR was performed using the miScript SYBR Green PCR Kit (Qiagen, 218073), and samples were amplified for 50 cycles as follows: 95°C for 15 s, 55°C for 30 s, and 72°C for 30 s. Small nuclear RNA (RNU6-6P RNA; Qiagen, MS00033740) was used for the normalization of the expression of miR-155-3p and miR-155-5p. The primer sequences used are shown in **Supplementary Table 2**.

### Transient transfection

BMDMs cultured in 48-well cell culture plates were transiently transfected with a miRNA mimic negative control (20 nM), miR-155-5p mimic (20 nM), miRNA inhibitor negative control (100 nM), or miR-155-5p inhibitor (100 nM) using the Lipofectamine 3000 Transfection Kit (Invitrogen, L3000-008) according to the manufacturer’s instructions. Genolution (Seoul, South Korea) provided the miR-155-5p mimic (5′-UUAAUGCUAAUUGUGAUAGGGGU-3′) and miR-155-5p inhibitor (5′-ACCCCUAUCACAAUUAGCAUUAA-3′), and Ambion (Austin, TX, USA) provided the miRNA mimic negative control (4464058) and inhibitor negative control (4464076).

### Colony-forming unit assay

BMDMs cultured in 48-well cell culture plates were transiently transfected with miRNA inhibitor negative control or miR-155-5p inhibitor before infecting with Mtb H37Rv at a multiplicity of infection (MOI) of 3 for 4 h. The infected cells were washed with PBS to remove extracellular bacteria and further incubated in the fresh medium for the indicated periods. Cells were then lysed in sterile distilled water for 30 min, serially diluted with PBS, and plated on the Middlebrook 7H10 agar plates containing OADC. Plates were incubated for 2-3 weeks at 37°C and colonies were enumerated to assess intracellular bacterial viability.

### *In-vivo* analysis with recombinant *M. smegmatis* strains

Frozen bacterial cells were centrifuged after thawing, and the pellet was resuspended in PBST. After anesthetizing C3HeB/FeJ mice, 1×10^6^ CFU/mouse of Ms_Vec or Ms_AcpM were inoculated intranasally. At the indicated times after infection, mice were euthanized and the lungs were collected to assess the bacterial burden. Lung tissues were homogenized using a tissue homogenizer (Omni International Inc., Warrenton, VA, USA) in PBST. Serial dilutions of the homogenates were planted in 7H10 agar plates, and colonies were counted after 3-4 days of incubation at 37°C.

### Statistical analysis

All of the experiments were repeated as indicated in figure legends, with consistent results. An unpaired Student’s t-test was used to determine the significance of differences between two groups, and an one-way analysis of variance (ANOVA) followed by Tukey’s multiple comparison test was used to determine the significance of differences among three or more groups using Prism^®^ software version 8 (GraphPad Software, San Diego, CA, USA). Data are expressed as means ± standard deviation (SD) or standard error of the mean (SEM); statistical significance was defined as *p < 0.05, **p < 0.01, and ***p < 0.001.

## Results

### AcpM inhibits TFEB expression and its nuclear translocation

**Figure 1:**
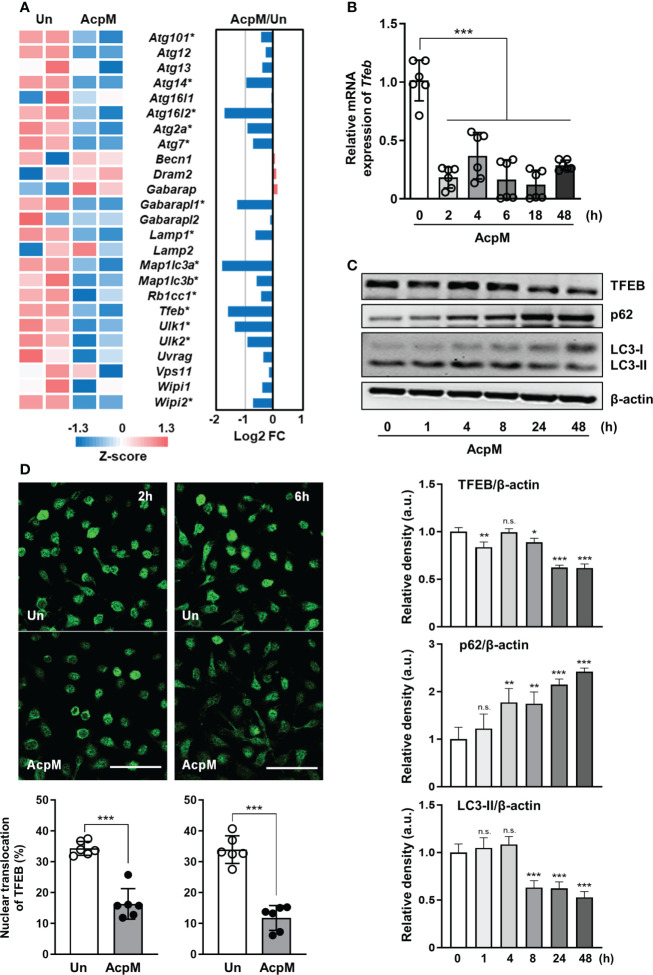
AcpM suppresses TFEB expression and its nuclear translocation. **(A)** A heatmap and a bar graph showing the expression of autophagy-associated genes in the AcpM-treated (AcpM, 10 μg/ml for 18 h) and untreated (Un) BMDMs. The left panel heatmap shows relative expression levels for each gene with Z-scores. The bar graph in the right panel depicts the fold change (FC). Gene names with an asterisk indicate statistical significance (FDR < 0.01). **(B, C)** BMDMs were treated with recombinant AcpM (10 μg/ml) for indicated times, and the harvested cells were subjected to either qRT-PCR analysis to measure *Tfeb* mRNA gene expression **(B)** or immunoblot analysis to measure TFEB protein expression **(C)**. One representative image, (**C**, upper panel) and the densitometric analysis (**C**, lower panel) of immunoblots were presented. **(D)** BMDMs treated with recombinant AcpM (10 μg/ml) for 2 or 6 h were harvested and stained with TFEB (green). Then the cells were subjected to confocal microscopy. Representative confocal images (Scale bar: 50 μm) from each group were presented. Statistical analysis was determined with an unpaired *t*-test or one-way ANOVA and presented as means ± SD from at least three independent experiments performed. *p < 0.05; **p < 0.01; ***p < 0.001. a.u., arbitrary unit; n.s., not significant; Un, untreated; AcpM, AcpM-treated.

To find the key molecule governing the host defense in AcpM-treated BMDMs, mRNA-seq analysis was performed (**Figure 1A**; **Supplementary Table 3**). Several autophagy-related genes, including *Tfeb*, were significantly downregulated in AcpM-treated BMDMs (AcpM) when compared to untreated cells (Un) (**Figure 1A**). Since TFEB is known to play a pivotal role in the regulation of lysosomal biogenesis and autophagy (24), qRT-PCR and western blot analysis were conducted to confirm its relative expression. Over time, AcpM treatment reduced the gene (**Figure 1B**) and protein (**Figure 1C**) levels of TFEB. Furthermore, AcpM treatment effectively suppressed the nuclear translocation of TFEB. The degree of TFEB in the nucleus reduced at early time points after AcpM addition in BMDMs, as shown by confocal images with TFEB staining in green (**Figure 1D**).

### AcpM suppresses the expression of numerous autophagy and lysosomal genes in the TFEB downstream pathway

**Figure 2:**
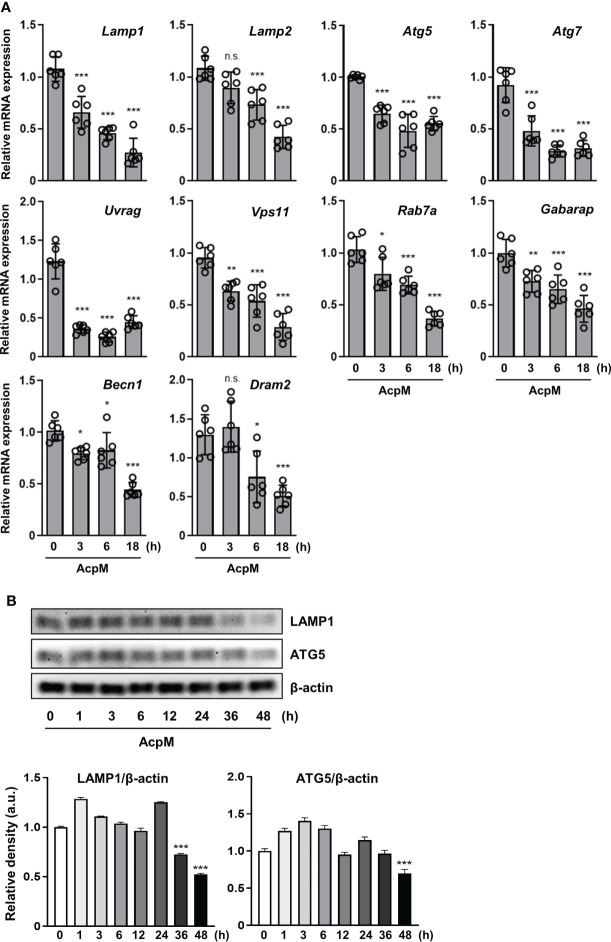
AcpM suppresses various autophagy and lysosomal genes. **(A)** BMDMs were treated with recombinant AcpM (10 μg/ml) for the indicated times. Total RNAs extracted from the cells were then subjected to qRT-PCR analysis to measure the expression of autophagic/lysosomal genes. **(B)** BMDMs treated with recombinant AcpM (10 μg/ml) for the indicated times were harvested, lysed, and subjected to immunoblot analysis to measure the LAMP1 and ATG5 expression. The representative image (upper panel) and the densitometric analysis (lower panel) of protein bands were presented. Statistical analysis was determined with one-way ANOVA and presented as means ± SD from at least three independent experiments performed. *p < 0.05; **p < 0.01; ***p < 0.001. a.u., arbitrary unit; n.s., not significant.

TFEB enters the nucleus to function as a transcription factor inducing lysosomal biogenesis. Since AcpM blocks its nuclear translocation (**Figure 1D**), various genes related to autophagy or lysosomal activity were thought to decrease with AcpM treatment in BMDMs. In detail, AcpM treatment significantly reduced the levels of *Lamp1*, *Lamp2*, autophagy-related gene 5 (*Atg5*), *Atg 7*, and several *Tfeb* downstream genes such as *Uvrag* and *Vps11* over time (**Figure 2A**). AcpM also significantly suppressed the expression of *Rap7a*, *Gabarap*, *Beclin-1* (*Becn1*), and damage-regulated autophagy modulator 2 (*Dram2*) at most time points (**Figure 2A**). Moreover, both LAMP1 and ATG5 protein levels in BMDMs were significantly reduced at 48 h after AcpM treatment (**Figure 2B**). Collectively, AcpM addition blocks nuclear translocation of TFEB, thereby downregulating the expression of various autophagy and lysosomal genes in BMDMs.

### AcpM inhibits LC3-II/LC3-I ratio, but does not affect autophagic flux in murine macrophages

**Figure 3:**
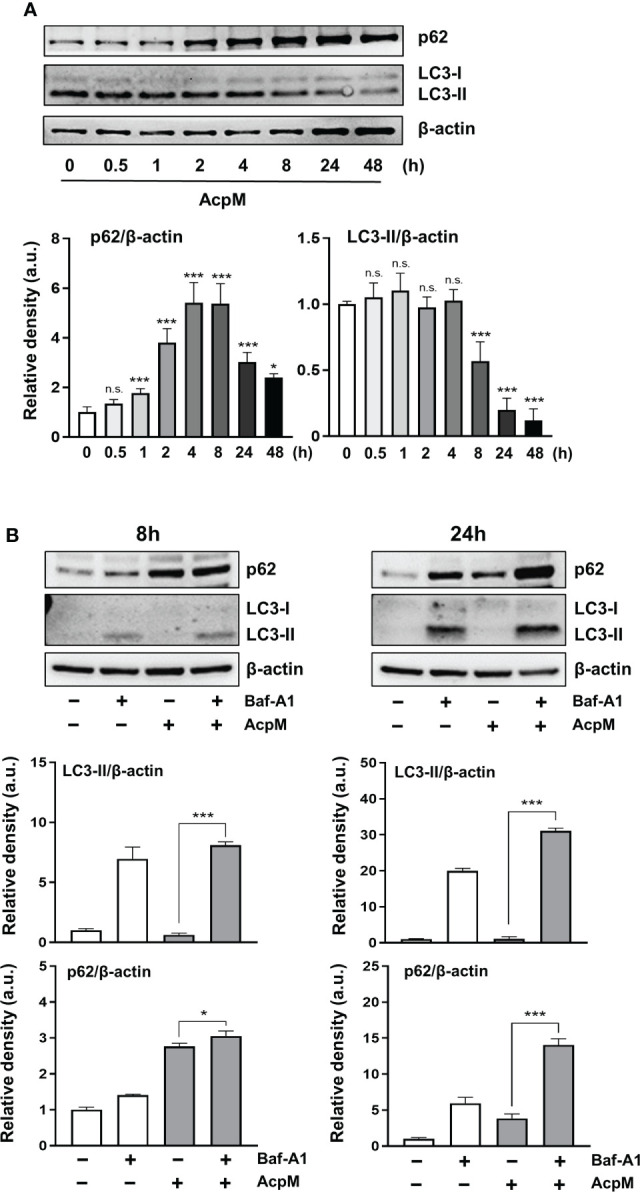
AcpM has no effect on autophagic flux in macrophages. **(A)** BMDMs were treated with recombinant AcpM (10 μg/ml) for the indicated times and the cell lysates were subjected to immunoblot analysis. One representative image (upper panel) and the densitometric analysis of the protein bands (lower panel) were presented. **(B)** BMDMs were pretreated with or without Baf-A1 (50 nM) for 1 h and then followed by AcpM (10 μg/ml) treatment. After 8h or 24 h, cells were harvested and subjected to immunoblot analysis with cell lysates. One representative image (upper panel) and the densitometric analysis (lower panel) of immunoblots were presented. Statistical analysis was determined with an one-way ANOVA and presented as means ± SD from at least three independent experiments performed. *p < 0.05; ***p < 0.001. a.u., arbitrary unit; n.s., not significant.

To determine whether AcpM affected autophagy in murine BMDMs, p62 and LC3 levels were validated by western blotting. AcpM treatment increased p62 while decreasing the LC3-II band over time (**Figure 3A**). To confirm the effect of AcpM in autophagic flux, the vacuolar type H^+^-ATPase (V-ATPase) inhibitor bafilomycin A1 (Baf-A1) was used. Baf-A1 was added 1 h before AcpM treatment to inhibit the lysosomal activity. After 8 h and 24 h, LC3-II bands in the AcpM-treated cells showed a significant difference in Baf-A1-untreated and -treated conditions, indicating that AcpM had no effect on the basal autophagic flux (**Figure 3B**). Furthermore, at 24 h after AcpM treatment, p62 levels were higher in Baf-A1-treated cells than in Baf-A1-untreated cells, implying that p62 accumulation in AcpM-treated conditions is not due to a block in autophagic flux. These findings indicate that, while AcpM inhibits LC3-II/LC3-I ratio over time, it has no effect on autophagic flux in BMDMs.

### AcpM suppresses phagosomal maturation of Mtb during infection

**Figure 4:**
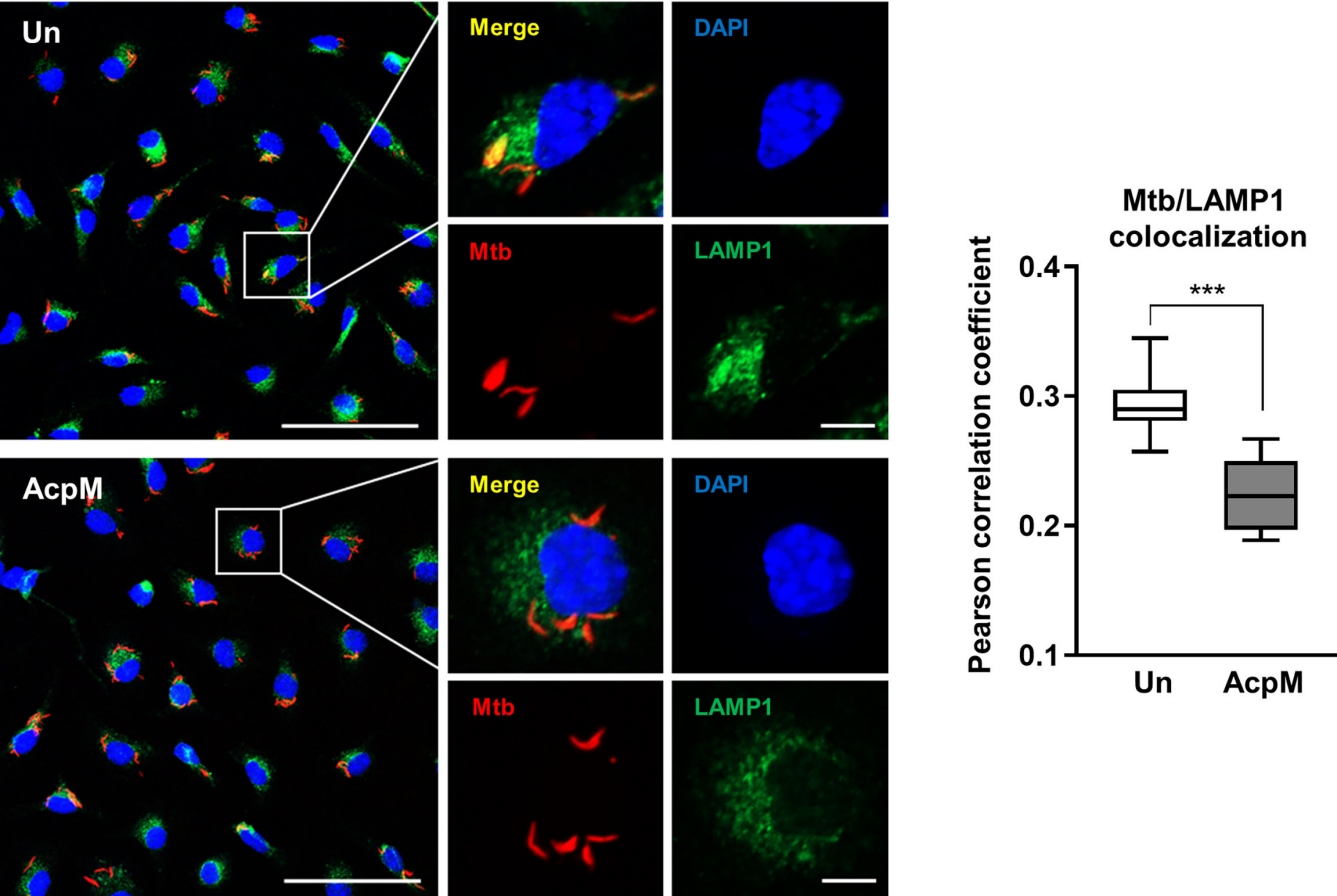
AcpM inhibits phagosome-lysosome fusion of Mtb. BMDMs were infected with Mtb-ERFP (MOI 5) for 4 h and then incubated with or without AcpM (10 μg/ml) in the freshly changed media for 4 h. Cells were stained with anti-LAMP1 (green) antibody and DAPI (blue) to visualize fluorescent images using Zeiss LSM-900 confocal microscopy (Scale bar: 50 μm for field views, 5 μm for single cell images). The colocalization rates between Mtb-ERFP and LAMP1 were assessed by calculating Pearson correlation coefficient from 12-15 field images (at least 80 cells per image). Statistical analysis was determined with an unpaired *t*-test and presented as means ± SD from at least three independent experiments performed. ***p < 0.001. Un, untreated; AcpM, AcpM-treated.

The next question was whether adding AcpM protein to Mtb-infected macrophages would affect phagosomal maturation. BMDMs were infected with an Mtb-ERFP strain, which was followed by AcpM treatment in fresh media. The cells were then stained with LAMP1 antibody to visualize lysosomes in confocal microscopy analysis. The colocalizing rate between Mtb and LAMP1 was significantly lower in the AcpM-treated conditions than in the untreated group (**Figure 4**). Therefore, AcpM helps Mtb circumvent phagosomal maturation by blocking phagosome and lysosome fusion.

### AcpM induces Akt-mTOR signaling *via* upregulating SHIP1-targeting miR-155-5p expression

**Figure 5:**
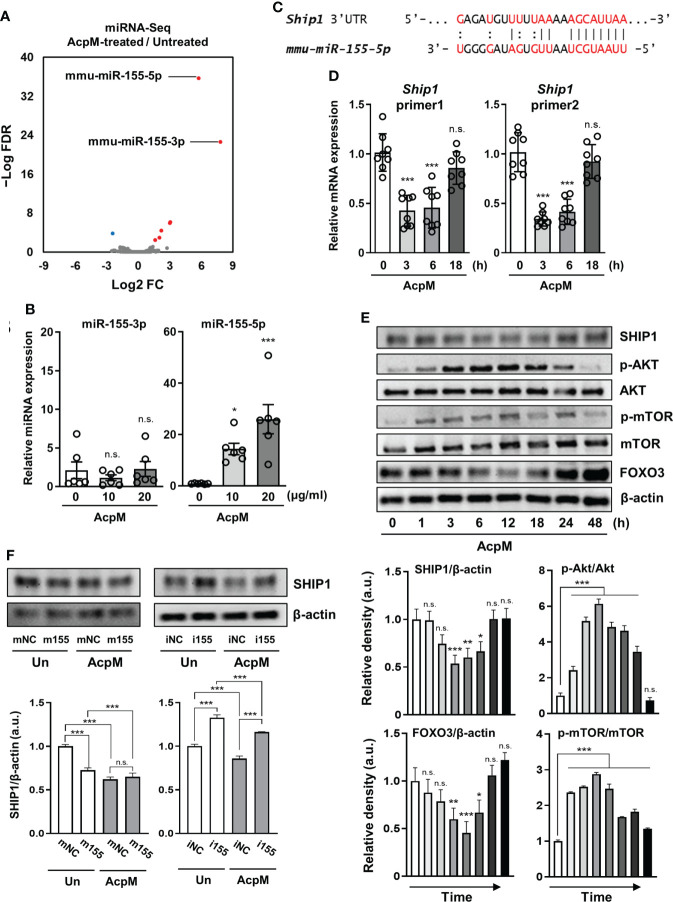
AcpM suppresses SHIP1 by increasing miR-155 expression. **(A)** A volcano plot representing differentially expressed miRNAs with the log2-fold change (FC) plotted against the negative log10 false discovery rate (FDR) for the AcpM-treated group compared to the untreated group. Red and blue dots indicate upregulated and downregulated genes, respectively. **(B)** BMDMs were treated with recombinant AcpM (10 or 20 μg/ml) for 8 h and the cell lysates were subjected to qRT-PCR analysis to measure the miR-155-3p and miR-155-5p expression. **(C)** The 3′ UTR of *ship1* mRNA is shown schematically, along with the relative location of the mouse miR-155-5p binding site. **(D**, **E)** BMDMs were treated with recombinant AcpM (10 μg/ml) for indicated times, and the harvested cells were subjected to either qRT-PCR analysis to determine the gene expression of *Ship1* **(D)** or immunoblot analysis to measure the expression of SHIP1 and SHIP1-downstream signaling molecules **(E)**. The representative image (**E**, upper panel) and the densitometric analysis (**E**, lower panel) of protein bands were presented. **(F)** BMDMs were transfected with mNC, m155, iNC, or i155, then further treated for 8 h with recombinant AcpM (10 μg/ml). Cells were lysed and subjected to immunoblot analysis to determine the SHIP1 protein level. The representative image (upper panel) and the densitometric analysis (lower panel) of SHIP1 bands were presented. Statistical analysis was determined with an unpaired *t*-test or one-way ANOVA and presented as means ± SD from at least three independent experiments performed. *p < 0.05; **p < 0.01; ***p < 0.001. a.u., arbitrary unit; n.s., not significant; mNC, negative control of miR-155-5p mimic; m155, miR-155-5p mimic; iNC, negative control of miR-155-5p inhibitor; i155, miR-155-5p inhibitor. Un, untreated; AcpM, AcpM-treated.

Previous studies have highlighted the importance of miRNAs in the regulation of host immune response (25–27). To see if AcpM was involved in the increase of specific miRNAs, miRNA-seq analysis was performed. The expression rates of miRNA-155p-3p and miRNA-155p-5p were the highest among the miRNAs that showed a significant change in the miRNA-seq analysis of AcpM-treated BMDMs when compared to untreated cells (**Figure 5A**, **Supplementary Table 4**). However, the qRT-PCR analysis revealed that miR-155-5p increased more than tenfold with increasing AcpM concentration in BMDMs, while miR-155-3p showed no significant change (**Figure 5B**). Previous studies showed that SHIP1 prevented Akt phosphorylation, thus blocking the Akt-mTOR pathway (18, 28). Also, as miR-155 was shown to target SHIP1 from an earlier study (**Figure 5C**) (29), the gene expression and protein amount of SHIP1 was investigated under AcpM treatment in BMDMs. At 3 and 6 h-post AcpM treatment, *Ship1* expressions analyzed with two different primers were significantly suppressed (**Figure 5D**). In western blot analysis, total SHIP1 expression was also significantly reduced from 3 to 18 h after AcpM administration, which was accompanied by an increase in phosphorylation of Akt and mTOR (**Figure 5E**). Along with increased Akt phosphorylation, there was also a reduction in FOXO3 levels (**Figure 5E**). To further demonstrate the ability of AcpM-induced miR-155-5p to regulate SHIP1 expression, miR-155-5p mimic and inhibitor (m155 and i155, respectively), as well as negative controls of miRNA mimic and inhibitor (mNC and iNC, respectively), were transfected into BMDMs. It was discovered that either m155 transfection or AcpM addition suppressed SHIP1 effectively and that i155 transfection could counteract AcpM-induced miR-155-5p expression and restore SHIP1 levels (**Figure 5F**). Overall, these findings suggest that AcpM-induced miR-155-5p plays a role in Akt-mTOR activation by targeting SHIP1.

### AcpM promotes Mtb intracellular survival by inducing the expression of miR-155-5p

**Figure 6:**
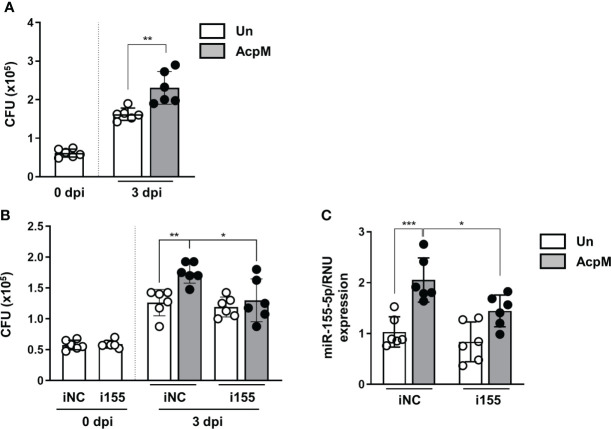
AcpM increases intracellular survival of Mtb by miR-155 upregulation. **(A)** BMDMs were infected with Mtb H37Rv (MOI 3) for 4 h and treated with recombinant AcpM (10 μg/ml) in the fresh media. After 3 days, cells were lysed and subjected to a CFU assay to explore the intracellular survival of Mtb. **(B**, **C)** BMDMs were transfected with either iNC or i155, then infected with Mtb H37Rv (MOI 3) for 4 h before treating recombinant AcpM (10 g/ml) in fresh media. Cells were lysed and subjected to CFU assay at the indicated times **(B)** or qRT-PCR after 18 h **(C)**. Statistical analysis was determined with an unpaired *t*-test and presented as means ± SD from at least three independent experiments performed. *p < 0.05; **p < 0.01; ***p < 0.001. iNC, negative control of miR-155-5p inhibitor; i155, miR-155-5p inhibitor. Un, untreated; AcpM, AcpM-treated.

Because AcpM inhibited Mtb fusion with lysosomes (**Figure 4**), Mtb ICS was thought to be increased. As expected, the Mtb CFU level was significantly higher in BMDMs 3 days after AcpM treatment than in the untreated group (Un) (**Figure 6A**). Furthermore, when i155-transfected groups were compared to iNC-transfected groups, CFU level in the AcpM-treated groups was significantly reduced (**Figure 6B**). Relative miR-155-5p expression in the same experimental settings as in **Figure 6B** revealed a positive correlation between the miR-155-5p and the Mtb CFU levels in BMDMs (**Figure 6C**). According to the findings, AcpM is thought to promote Mtb survival in BMDMs by upregulating miR-155-5p expression.

### AcpM overexpression enhances *in-vivo* survival of *M. smegmatis* in C3HeB/FeJ mice

**Figure 7:**
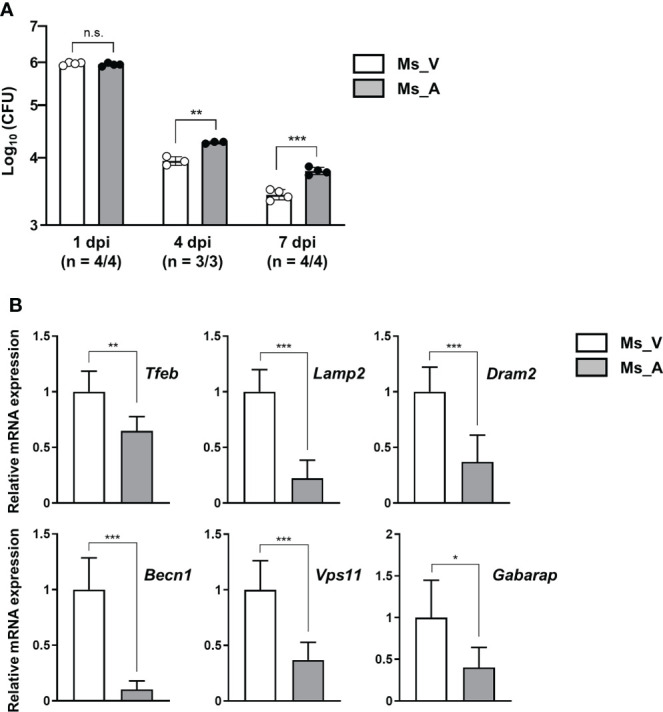
AcpM overexpression increases the survival of *M. smegmatis in-vivo*. **(A)** C3HeB/FeJ mice (n = 22) were intranasally infected with recombinant *M. smegmatis* strains Ms_AcpM (n = 11) or Ms_Vec (n = 11) and euthanized at the indicated times after infection (1, 4, or 7 dpi). The lungs were resected from mice to assess the bacterial burden by CFU assay. **(B)** Lung lysates from two randomly selected mice from each group were analyzed in triplicate using qRT-PCR to evaluate the expression level of autophagic/lysosomal genes at 7 dpi. Statistical significances were calculated with an unpaired *t*-test. Data are presented as mean ± SD. *P < 0.05; **P < 0.01; ***P < 0.001; a.u., arbitrary unit; n.s., not significant; CFU, colony-forming unit; dpi, days post-infection; Ms_V, Ms_Vec-infected; Ms_A, Ms_AcpM-infected.

To evaluate the effect of AcpM secretion *in-vivo*, recombinant *M. smegmatis* strains overexpressing AcpM (Ms_AcpM) and a vector plasmid carrying control (Ms_Vec) were used. C3HeB/FeJ mice were challenged with either Ms_Vec or Ms_AcpM *via* nasal route and sacrificed at 1, 4, and 7 days post-infection (dpi). One day after infection, there was no significant difference in CFU levels between lung lysates from two recombinant strains-infected mice, indicating that an equal amount of strains was properly administered through the nasal airways (**Figure 7A**). However, the viability of Ms_AcpM was significantly higher than that of Ms_Vec at 4 and 7 dpi (**Figure 7A**), suggesting that AcpM overexpression improves *M. smegmatis in-vivo* survival. Interestingly, qRT-PCR analysis of the samples obtained from the same mice revealed a decrease in several autophagy and lysosomal genes including *Tfeb* (**Figure 7B**). These data suggest that AcpM overexpression helps *M. smegmatis* survival in mouse lungs, possibly by altering TFEB downstream pathways as shown in murine macrophages.

## Discussion

**Figure 8:**
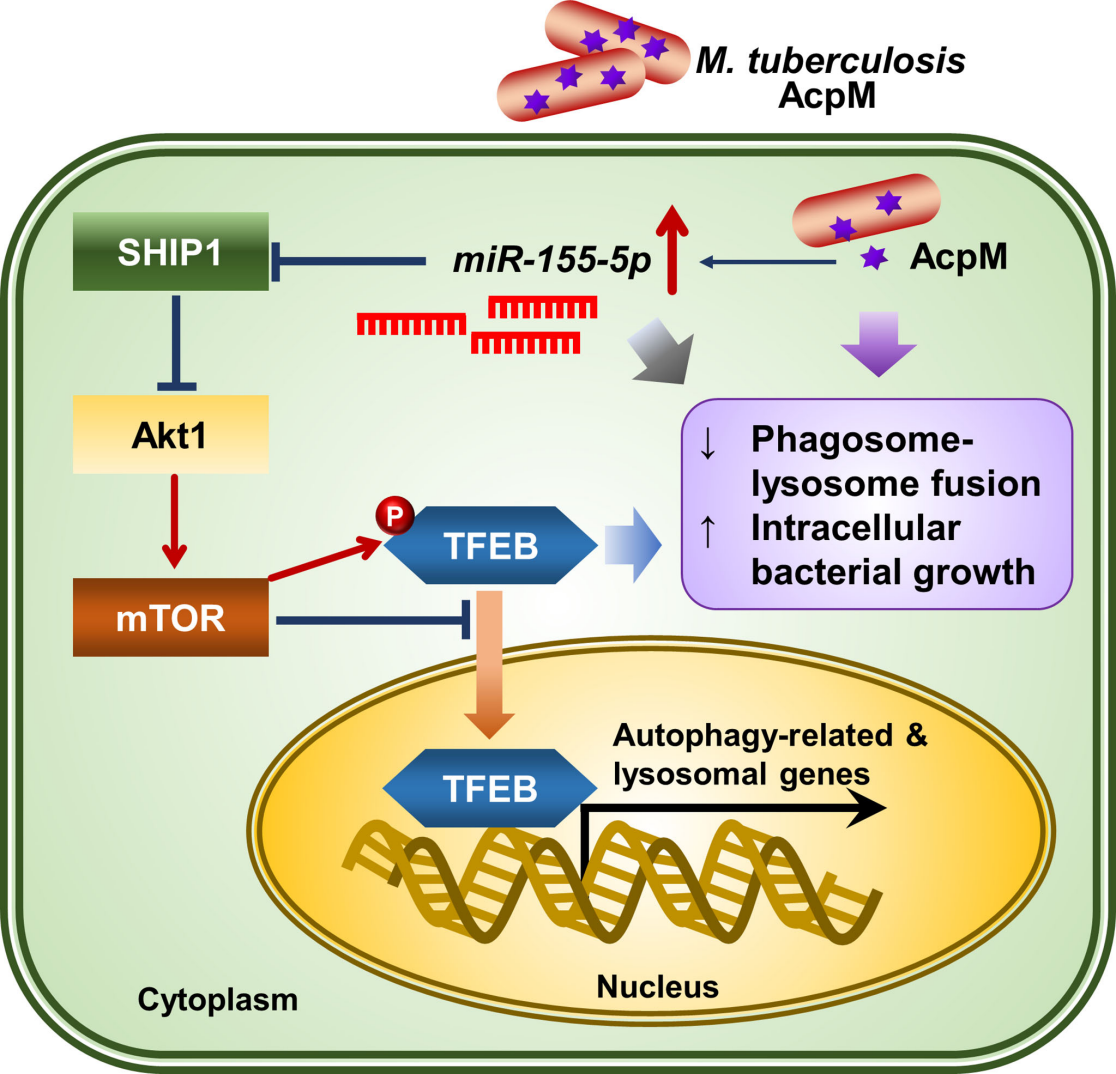
The proposed mechanism of action of AcpM in Mtb-infected macrophages. AcpM promotes the expression of miR-155, which targets SHIP1 to activate the Akt/mTOR pathway. The activated Akt/mTOR signaling pathway inhibits TFEB nuclear translocation and reduces the expression of autophagy and lysosomal genes, which is likely to induce antimicrobial defense in macrophages. AcpM also improves intracellular mycobacterial survival by inhibiting phagosome-lysosome fusion.

In this study, AcpM, an essential protein for Mtb survival and mycolic acid synthesis (30), was newly discovered as a mycobacterial effector for pathogenesis through blocking TFEB activation and increasing miR-155-5p expression. A schematic summary of the AcpM’s suggested mode of action was presented in **Figure 8**. Previously, the apoptosis inhibiting feature of AcpM was also described (19). In murine BMDM settings, AcpM did not directly affect autophagic flux, but significantly suppressed multiple autophagy gene expression, which may influence host defense pathways in an autophagy-independent manner. Importantly, we found that the mRNA and protein expression of LAMP1, which is regulated by TFEB (31), was down-regulated by AcpM, suggesting that AcpM affects lysosomal biogenesis during Mtb infection. In addition, our data highlights the AcpM function in the elevation of miR-155-5p, which was shown to target SHIP1 (29, 32, 33). Previous studies showed that SHIP1 plays an essential role in the activation of Akt pathway, thereby enhancing intracellular Mtb survival (18). In addition, miR-155 can target FOXO3 (34), which is associated with the gene expression of multiple autophagy-related genes such as *Atg5*, *Atg12*, *Becn1*, *Lc3* and *Bnip3* (35, 36). However, the role of Mtb-induced miR-155 expression in regulating host defense in the early stages of infection has sparked debate. Wang et al. reported that miR-155 induced autophagy to eliminate intracellular mycobacteria by targeting Ras homolog enriched in brain (Rheb) in RAW264.7 cells (37). Indeed, the miR-155 level is elevated in both Mtb-infected macrophages (37) and active TB patients (38). On the other hand, Rothchild et al. demonstrated that miR-155 promoted Mtb survival in BMDMs through targeting SHIP1 in the early stages of infection, even though it also activated Mtb-specific T cell function in the adaptive immune response to effectively reduce bacterial survival in the late stages of infection (28). Kumar et al. also discovered that overexpression of miR-155 reduced the expression of BTB and CNC homology 1 (BACH1) and SHIP1, allowing Mtb to survive in macrophages (18). These results show a partial correlation with ours that miR-155 favors mycobacterial survival in macrophages by targeting SHIP1-Akt axis. Although the role of miR-155 in host defense regulation varies depending on the host cell type or bacterial strain, it appears that miR-155 inhibits antimicrobial host defense in macrophages in the early stages of infection.

TFEB is known as a master regulator of lysosomal biogenesis (24). Previous research reported that the suppression of the Akt-mTOR pathway enhances nuclear translocation of TFEB to induce transcriptional activation of lysosomal and autophagy-related genes (39, 40). According to our findings, AcpM increased Akt and mTOR phosphorylation (**Figure 5E**) while decreasing TFEB expression and its nuclear translocation (**Figure 1**), which likely leads to the downregulation of autophagy and lysosomal genes (**Figure 2**). Recent studies showed that TFEB activation is critically involved in the regulatory node of antimicrobial responses against Mtb in macrophages (41–43). Importantly, we found that AcpM did not affect the induction of autophagy or activation of autophagic flux when treated with Baf-A1 in basal conditions at both 8 h and 24 h after AcpM treatment (**Figure 3**). Thus, the AcpM’s role in the suppression of antimicrobial responses against Mtb infection seems to be associated with the inhibition of TFEB, but not directly related to the suppression of autophagy. In addition, a recent study revealed that TFEB activation is required for the induction of mitochondrial itaconate synthesis to control intracellular bacterial growth (44, 45), suggesting the critical function of TFEB in terms of antimicrobial defense in macrophages. Future studies will clarify whether AcpM is involved in the regulation of immunometabolic remodeling in macrophages to further affect TFEB-induced antimicrobial responses during Mtb infection.

We also found that AcpM increased miR-155 production, which targets SHIP1 to prevent its negative regulation on Akt phosphorylation, resulting in the increased Mtb survival in host cells. Because AcpM-induced miR-155-5p upregulates the Akt/mTOR pathway by targeting SHIP1, it is supposed that miR-155-5p-mediated Akt/mTOR activation leads to the suppression of TFEB activation. Since the level of miR-155 is related to the virulence of infected mycobacterial strains (18, 37), the present data is important to show the function of AcpM as an inducer of miR-155 to further regulate the host protective responses during infection. In this regard, identifying other mycobacterial factors that stimulate miR-155 expression and elucidating the exact mechanism of how mycobacteria activate miR-155 production would help us better understand mycobacterial pathogenesis.

To further understand the function of AcpM during mycobacterial infection, an attempt was made to construct an AcpM-conditional knockout system using the Mtb H37Rv strain. However, we were unable to achieve it, most likely due to the AcpM’s essential role in Mtb survival. Thus, *M. smegmatis* strains, Ms_AcpM and Ms_Vec, were used to test if AcpM overexpression could increase the number of surviving bacteria in lung tissues of infected mice. Because *M. smegmatis* strains are non-pathogenic, they have little tolerance for the host’s innate immune system. To slow down the declining survival rate of recombinant *M. smegmatis* strains, an *in-vivo* challenge was conducted using C3HeB/FeJ mice (46). As a result, CFU levels of Ms_AcpM were significantly higher than that of Ms_Vec, implying that AcpM overexpression improves the survival of *M. smegmatis in-vivo* (**Figure 7A**). Thus, AcpM expressed in mycobacteria is likely to suppress the *tfeb* and *tfeb*-downstream autophagy-related gene expression in the lung tissues in the same way that recombinant AcpM protein does in macrophages.

Recently, a small molecule called “8918,” which selectively binds to PptT, was discovered to have anti-tuberculosis efficacy comparable to rifampin, a first-line anti-tuberculosis drug (17). In addition, a newly discovered Ppt hydrolase, PptH, which removes Ppt from AcpM, made Mtb more sensitive to 8918, even when PptT was only partially inhibited (17). Therefore, it’s possible to believe that Mtb virulence is influenced by the formation and maintenance of holo-AcpM. Finding small chemical compounds that can selectively target AcpM could be helpful in the development of new anti-mycobacterial drugs.

In summary, AcpM’s role in modulating antimicrobial host defense was revealed in this work. AcpM was discovered to effectively reduce TFEB nuclear translocation and downregulate the expression of autophagy and lysosomal genes in macrophages. In addition, AcpM-mediated miR-155-5p activated the Akt/mTOR pathway by targeting SHIP1. AcpM also improved intracellular mycobacterial survival by reducing phagosome-lysosome fusion. These findings highlight the importance of understanding host-pathogen interactions in the context of the Mtb virulence factors and provoke future studies targeting AcpM to expand the development of novel Mtb therapeutics.

## Data availability statement

All mRNA-seq and miRNA-seq data generated in this study are available through the NCBI Gene Expression Omnibus through accession numbers SRR18614842-SRR18614845 and SRR18615277-SRR18615280 under BioProject PRJNA823491.

## Ethics statement

The animal study was reviewed and approved by Institutional Research and Ethics Committee at Chungnam National University, School of Medicine (202009A-CNU-155; Daejeon, Korea).

## Author contributions

SP was in charge of the majority of the data processing and analysis. SP, KK, IK, YK, and H-JK carried out the experiments and data analysis. SP and SC constructed and purified the recombinant AcpM protein and the *M. smegmatis* strains used in this study. SP, KK, IK, and YK wrote the manuscript, which was then peer-reviewed by H-JK and E-KJ. SP and E-KJ guided and supervised the work. All authors contributed to the article and approved the submitted version.

## Funding

This work was supported by the National Research Foundation of Korea (NRF) grant funded by the Korean government (MSIT) (No. 2021R1C1C2006968 and 2017R1A5A2015385).

## Acknowledgments

We are grateful to Prof. Sung Jae Shin (Yonsei University, Korea) and Prof. Jin Kyung Kim (Keimyung University, Korea) for providing and harvesting Mtb-ERFP, respectively.

## Conflict of interest

The authors declare that the research was conducted in the absence of any commercial or financial relationships that could be construed as a potential conflict of interest.

## Publisher’s note

All claims expressed in this article are solely those of the authors and do not necessarily represent those of their affiliated organizations, or those of the publisher, the editors and the reviewers. Any product that may be evaluated in this article, or claim that may be made by its manufacturer, is not guaranteed or endorsed by the publisher.
