# Supplementary material for: Mycobacterial acyl carrier protein suppresses TFEB activation and upregulates miR-155 to inhibit host defense
Source: Front Immunol. 2022 Sep 28;13:946929. doi: 10.3389/fimmu.2022.946929 (PMC9559204; doi:10.3389/fimmu.2022.946929)
Supplement: Supplementary file 1 [file DataSheet_1.pdf]

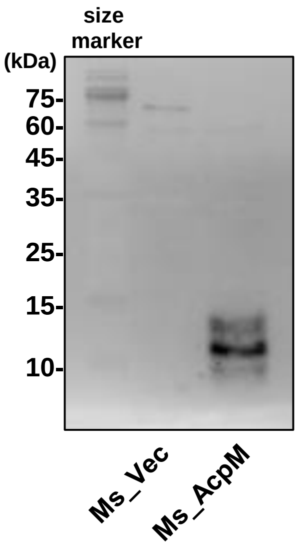

Supplementary Figure S1. AcpM expression in recombinant *M. smegmatis* strains. Ms_Vec and Ms_AcpM were cultured in 7H9 liquid medium supplemented with 10 % OADC, and the lysates were subjected to SDS-PAGE. Western blotting was performed to assess AcpM expression using an anti-AcpM antibody (1:100,000 dilution).

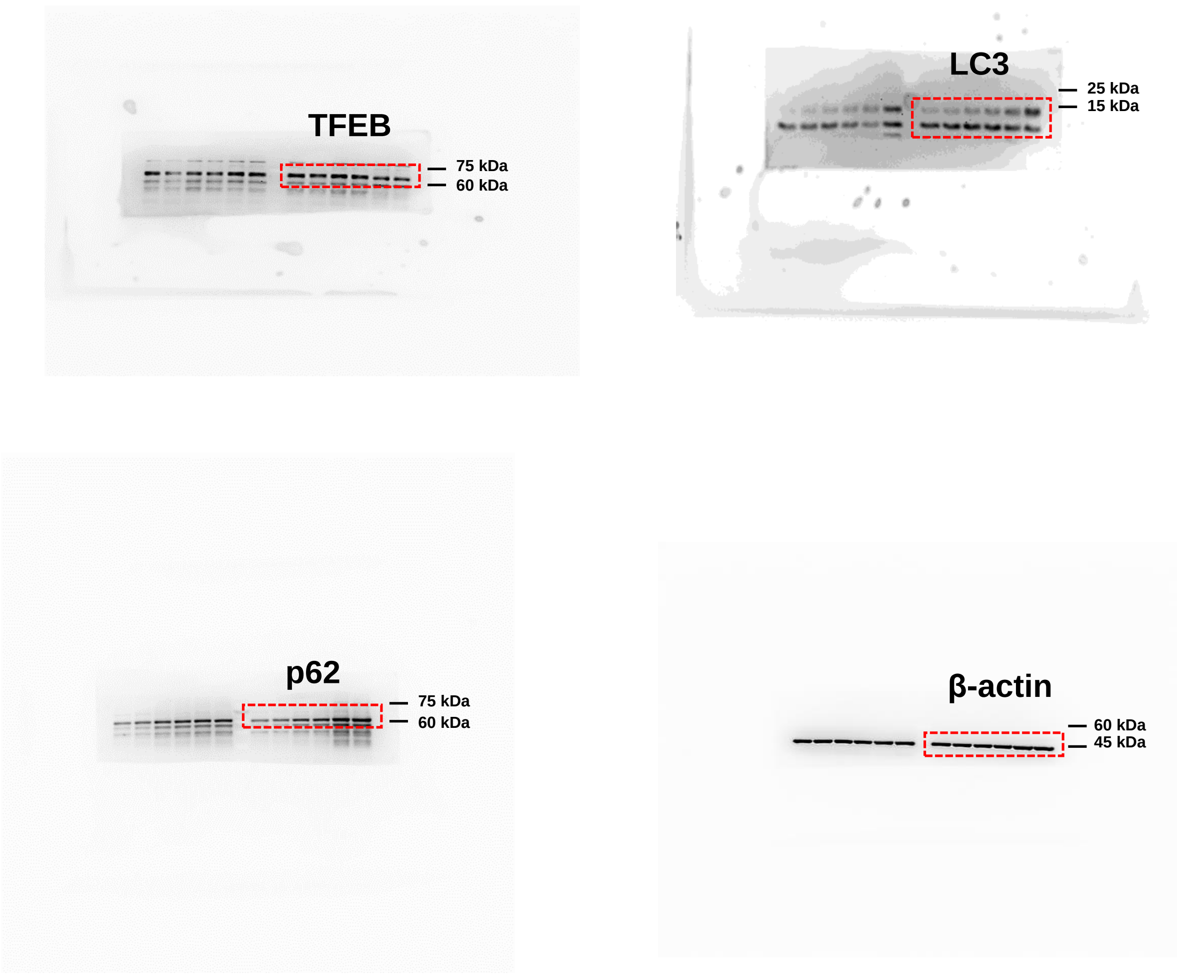

Supplementary Figure S2. Full-length western blots of the bands shown in Figure 1C.

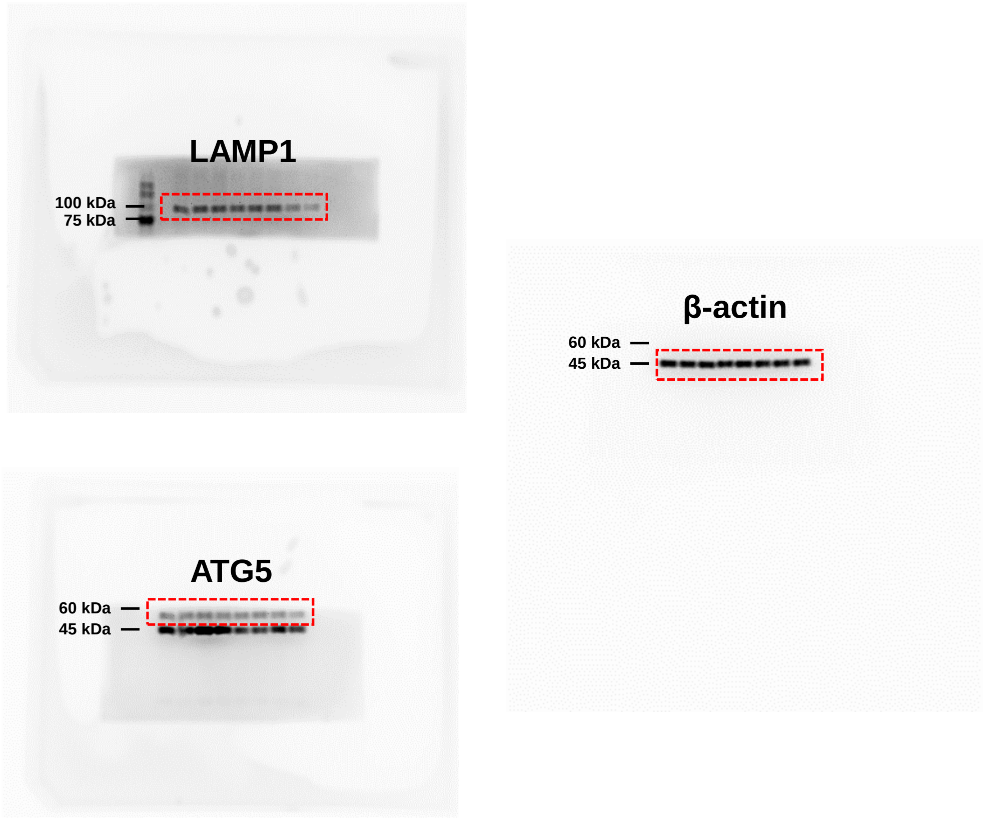

Supplementary Figure S3. Full-length western blots of the bands shown in Figure 2B.

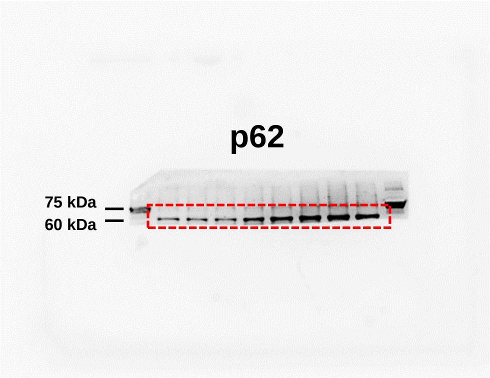

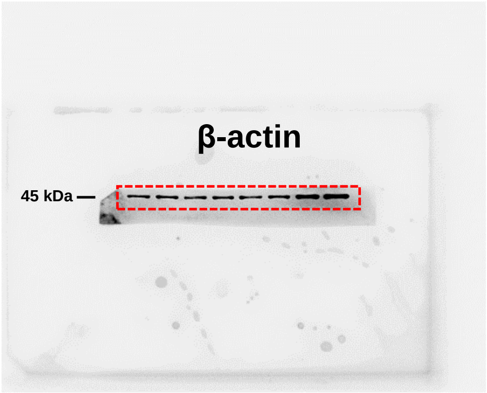

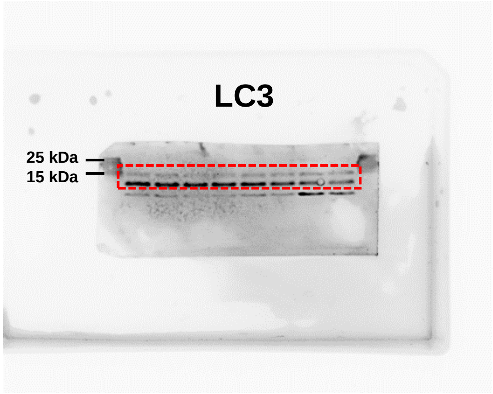

Supplementary Figure S4. Full-length western blots of the bands shown in Figure 3A.

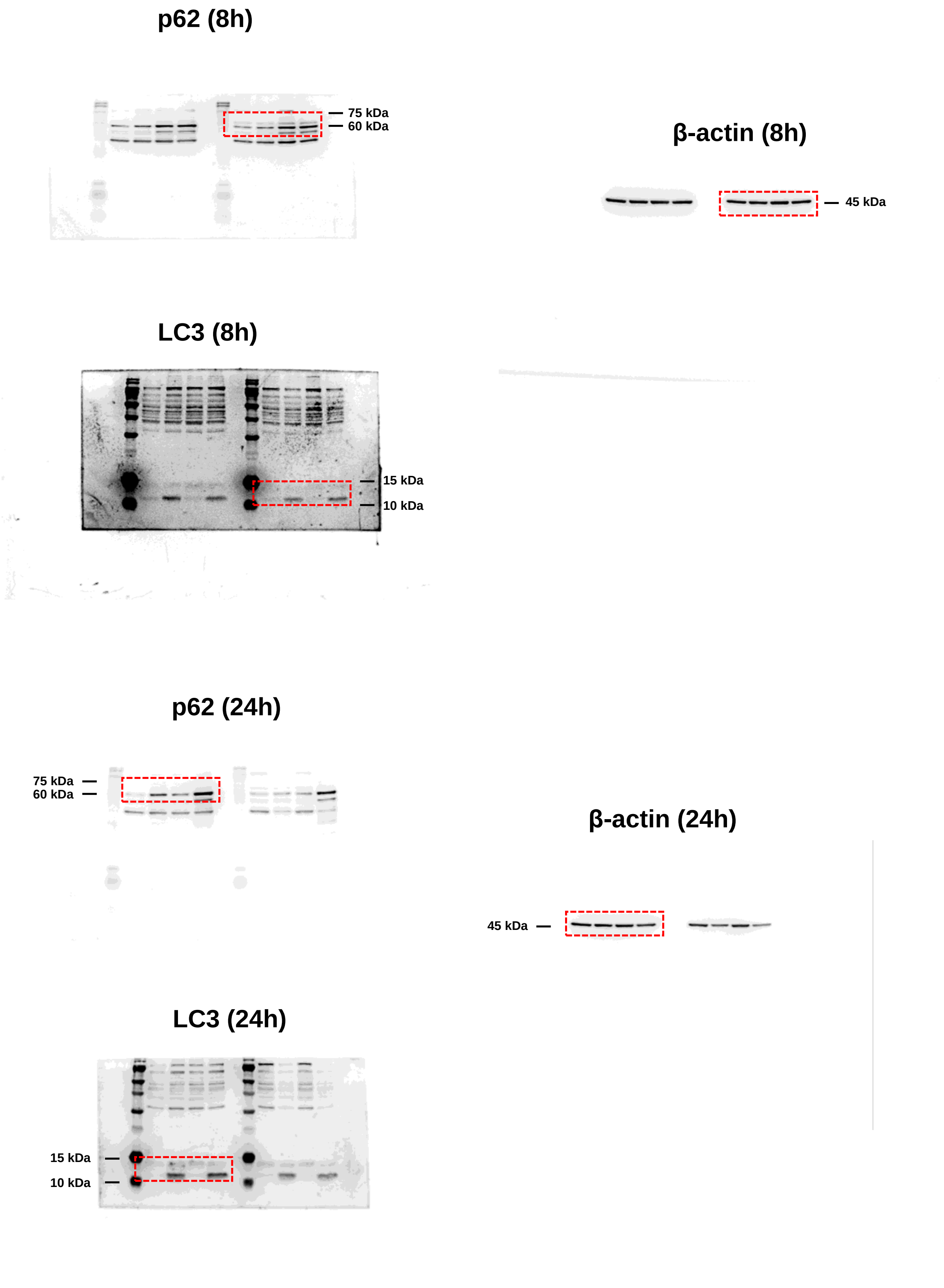

Supplementary Figure S5. Full-length western blots of the bands shown in Figure 3B.

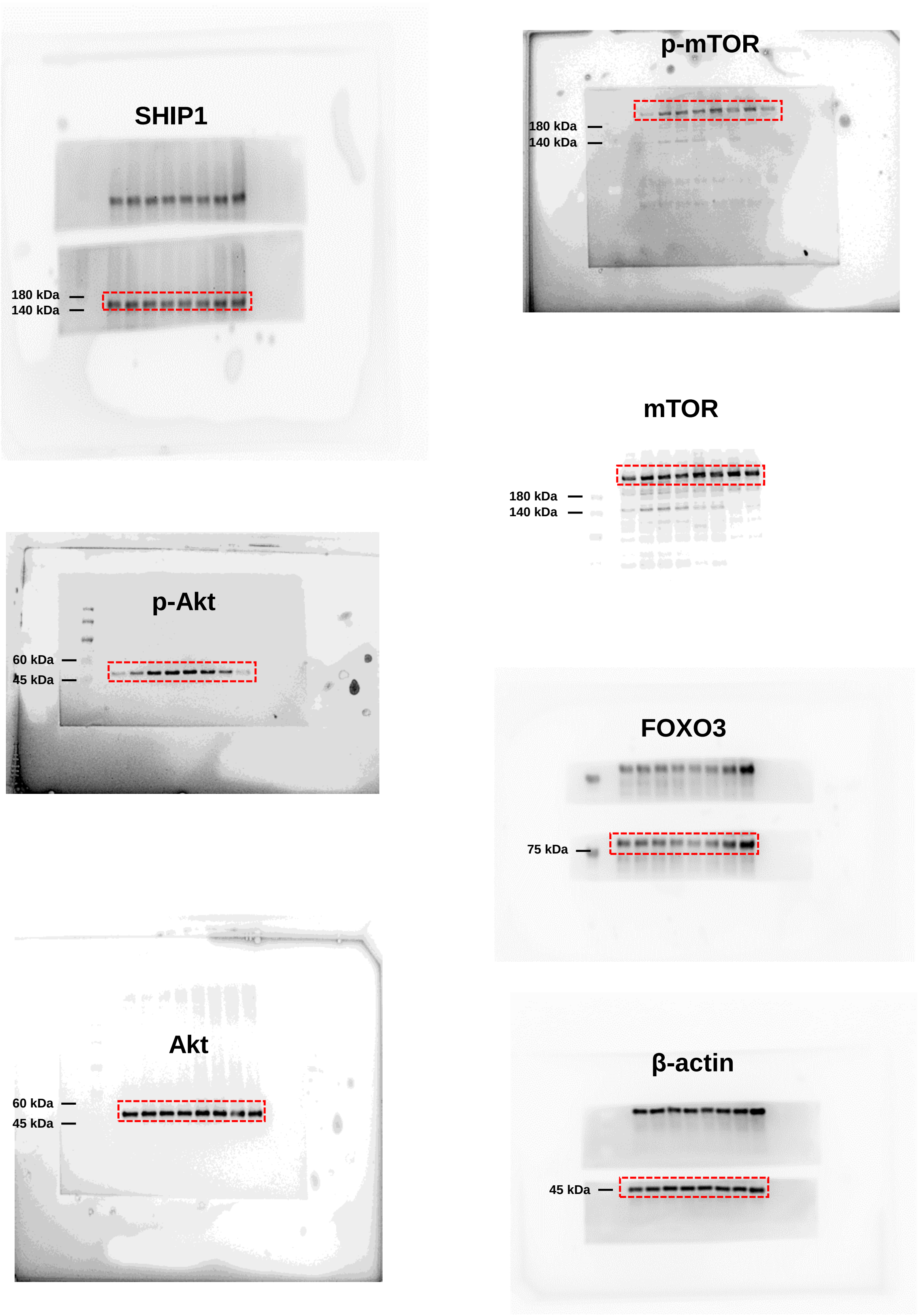

Supplementary Figure S6. Full-length western blots of the bands shown in Figure 5E.

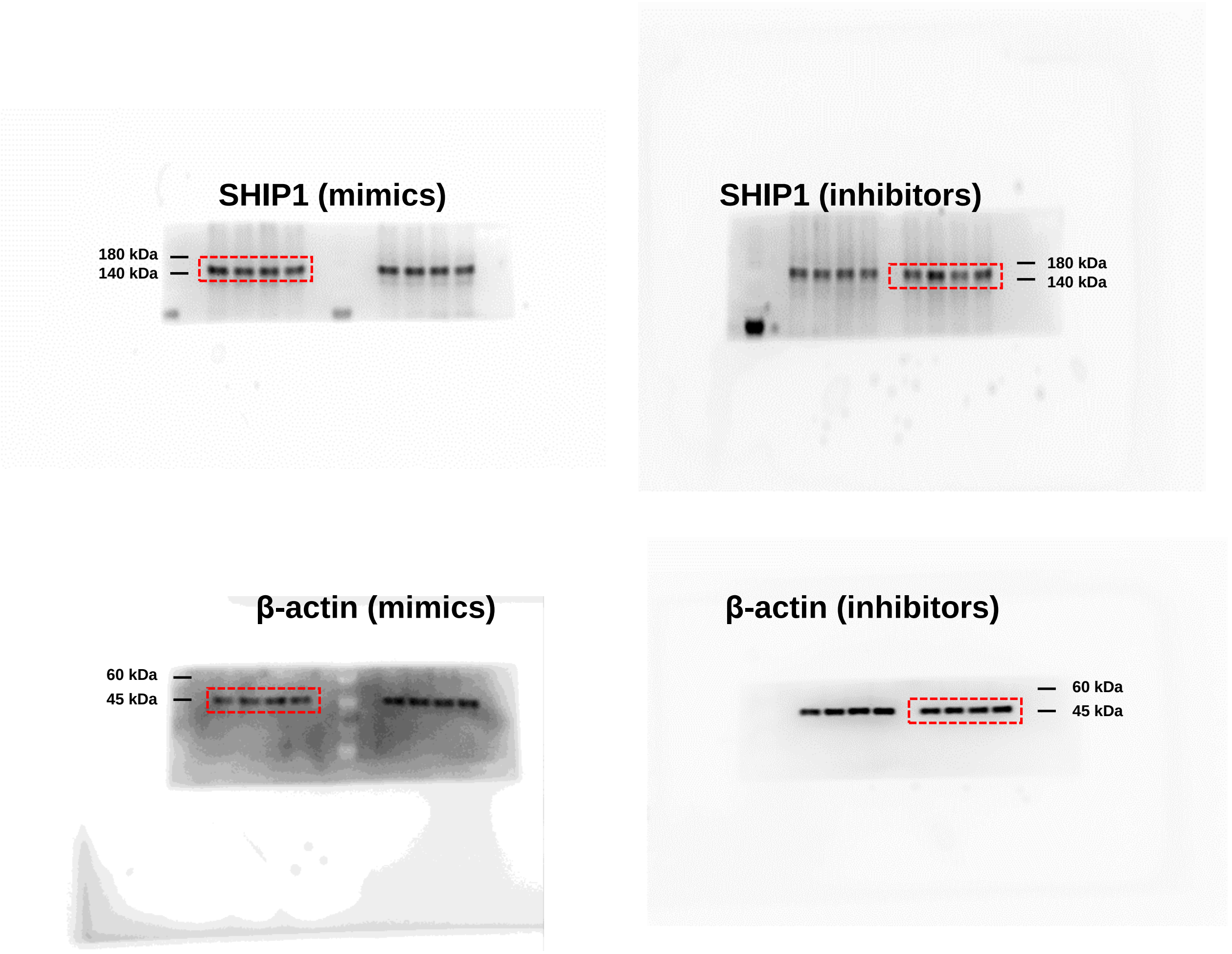

Supplementary Figure S7. Full-length western blots of the bands shown in Figure 5F.

Genes	Primer	Sequences
β-actin	Forward	5′-AGG GTG TAA AAC GCA GCT CA-3′
	Reverse	5′-CCA CCA TGT ACC CAG GCA TT-3′
Gapdh	Forward	5′-AAG ATG GTG ATG GGC TTC CCG-3′
	Reverse	5′-TGG CAA AGT GGA GAT TGT TGC C-3′
Tfeb	Forward	5′-CCA CCC CAG CCA TCA ACA C-3′
	Reverse	5′-CAG ACA GAT ACT CCC GAA CCT T-3′
Lamp1	Forward	5′-CAG CAC TCT TTG AGG TGA AAA AC-3′
	Reverse	5′-CCA TTC GCA GTC TCG TAG GTG-3′
Lamp2	Forward	5′-GAG CAG GTG CTT TCT GTG TCT AG-3′
	Reverse	5′-GCC TGA AAG ACC AGC ACC AAC T-3′
Atg5	Forward	5′-TGT GCT TCG AGA TGT GTG GT-3′
	Reverse	5′-ACC AAC GTC AAA TAG CTG AC-3′
Atg7	Forward	5′-CAG GAG ATT CAA CCA GAG AC-3′
	Reverse	5′-AGA TAC CAT CAA TTC CAC GG-3′
Uvrag	Forward	5′-GAC TTT GGA ATA ATG CCG GAT CG-3′
	Reverse	5′-CAG CCC ATC CAG GTA GAC TTT-3′
Vps11	Forward	5′-ATC GGC AGT CTC TGG CTA ATG C-3′
	Reverse	5′-GGA CCT TGA TGG CTG TCT CTA C-3′
Rab7a	Forward	5′-GAG CGG ACT TTC TGA CCA AGG A-3′
	Reverse	5′-CAA TCT GCA CCT CTG TAG AAG GC-3′
Gabarap	Forward	5′-AGG ACC ACC CCT TCG AGT ATC-3′
	Reverse	5′-GCA CAA GGT ACT TCC TCT TAT CC-3′
Becn1	Forward	5′-CAG CCT CTG AAA CTG GAC ACG A-3′
	Reverse	5′-CTC TCC TGA GTT AGC CTC TTC C-3′
Dram2	Forward	5′-CCC TGC ATT GCC TTA TAT CA-3′
	Reverse	5′-TGC TTG TAA CGA ACA TAC AT-3′
***Ship1* (primer1)**	Forward	5′-CCA GGG CAA GAT GAG GGA GA-3′
	Reverse	5′-GGA CCT CGG TTG GCA ATG TA-3′
***Ship1* (primer2)**	Forward	5′-GAG CGG GAT GAA TCC AGT GG-3′
	Reverse	5′-GGA CCT CGG TTG GCA ATG TA-3′

Supplementary Table 1. Mouse primer sequences used for mRNA qRT-PCR analysis

Gene symbol	Product number	Sequences
**mmu-miR-155-3p**	MS00024255	5′-CUC CUA CCU GUU AGC AUU AAC-3′
**mmu-miR-155-5p**	MS00001701	5′-UUA AUG CUA AUU GUG AUA GGG GU-3′

Supplementary Table 2. Mouse primer sequences used for miRNA qRT-PCR analysis

Gene symbol	Raw Counts				TMM normalized counts				FC Results		Z-score			
	Un1	Un2	AcpM1	AcpM2	Un1	Un2	AcpM1	AcpM2	Log2 FC	P-Value	Un1	Un2	AcpM1	AcpM2
Atg101	699	709	493	400	5.577	5.591	5.228	5.112	-0.414	0.002	0.821	0.878	-0.611	-1.087
Atg12	1275	1296	954	843	6.442	6.459	6.178	6.184	-0.270	0.017	0.811	0.919	-0.886	-0.844
Atg13	322	417	288	194	4.464	4.828	4.457	4.074	-0.380	0.053	0.027	1.210	0.002	-1.239
Atg14	432	434	203	177	4.886	4.886	3.956	3.943	-0.942	0.000	0.866	0.866	-0.854	-0.878
Atg16l1	339	394	319	287	4.538	4.747	4.603	4.635	-0.028	0.862	-1.062	1.329	-0.318	0.051
Atg16l2	315	393	118	67	4.433	4.743	3.183	2.564	-1.701	0.000	0.682	0.984	-0.532	-1.133
Atg2a	509	434	243	183	5.121	4.886	4.213	3.991	-0.905	0.000	1.059	0.620	-0.632	-1.046
Atg7	1109	1025	592	527	6.242	6.121	5.491	5.508	-0.685	0.000	1.010	0.708	-0.881	-0.838
Becn1	1505	1384	1345	1171	6.681	6.554	6.672	6.657	0.046	0.686	0.678	-1.479	0.527	0.274
Dram2	864	983	875	790	5.882	6.061	6.053	6.091	0.098	0.432	-1.478	0.418	0.332	0.728
Gabarap	3649	3584	3655	3080	7.958	7.925	8.113	8.051	0.141	0.208	-0.628	-1.006	1.177	0.457
Gabarapl1	1920	2268	806	664	7.032	7.266	5.935	5.841	-1.267	0.000	0.699	1.016	-0.793	-0.922
Gabarapl2	752	635	580	513	5.682	5.433	5.462	5.469	-0.098	0.478	1.486	-0.686	-0.434	-0.366
Lamp1	37236	35806	22436	18415	11.308	11.245	10.731	10.631	-0.596	0.000	0.948	0.767	-0.714	-1.001
Lamp2	5578	5968	5445	4535	8.570	8.661	8.688	8.609	0.033	0.774	-1.178	0.543	1.064	-0.430
Map1lc3a	688	685	213	130	5.554	5.542	4.025	3.503	-1.765	0.000	0.854	0.842	-0.600	-1.096
Map1lc3b	2107	2520	1352	1275	7.166	7.417	6.680	6.780	-0.567	0.000	0.453	1.187	-0.966	-0.674
Rb1cc1	1564	1636	987	1028	6.737	6.795	6.227	6.470	-0.414	0.001	0.686	0.909	-1.262	-0.333
Tfeb	566	589	197	133	5.274	5.324	3.913	3.536	-1.568	0.000	0.826	0.881	-0.649	-1.058
Ulk1	1156	901	335	359	6.301	5.936	4.673	4.957	-1.314	0.000	1.075	0.605	-1.023	-0.657
Ulk2	923	804	370	420	5.977	5.772	4.816	5.182	-0.873	0.000	1.012	0.628	-1.163	-0.477
Uvrag	763	653	475	479	5.703	5.473	5.175	5.371	-0.319	0.026	1.240	0.193	-1.162	-0.270
Vps11	329	373	315	220	4.495	4.668	4.585	4.254	-0.154	0.383	-0.032	0.938	0.471	-1.377
Wipi1	233	297	159	184	4.001	4.342	3.607	3.999	-0.372	0.082	0.046	1.181	-1.266	0.038
Wipi2	394	395	214	199	4.754	4.751	4.031	4.111	-0.686	0.000	0.867	0.859	-0.964	-0.762

Supplementary Table 3. The mRNA-seq analysis of autophagy-associated genes in AcpM-untreated and -treated BMDMs. TMM, trimmed mean of M values; FC, fold-change; P-Value, the two-sided p-value.

Gene symbol	Log2 FC	Log2 CPM	LR	P-Value	FDR
**mmu-miR-155-5p**	5.762019411	10.69419868	171.8078868	2.98E-39	1.79E-36
**mmu-miR-155-3p**	7.831204778	4.529125085	110.2091364	8.82E-26	2.65E-23
**mmu-let-7e-3p**	3.061301207	5.208937705	34.83407284	3.59E-09	7.19E-07
**mmu-miR-125a-3p**	2.993497478	7.690496292	33.39673438	7.52E-09	1.13E-06
**mmu-let-7c-1-3p**	2.189906116	5.765881991	25.9756277	3.46E-07	4.16E-05
**mmu-miR-7033-5p**	-2.436683069	3.436142483	23.01741231	1.61E-06	0.000160808
**mmu-miR-146a-3p**	2.008385218	4.861550059	19.1218872	1.23E-05	0.00105287
**mmu-miR-99b-3p**	1.617712221	8.10575209	16.32169347	5.34E-05	0.004015323
**mmu-let-7k**	1.373244356	5.522023762	10.61693683	0.001120565	0.074828854
**mmu-miR-144-3p**	-1.718653572	3.793475305	9.723177315	0.001819587	0.109357167
**mmu-miR-690**	-1.553319247	4.327208398	9.186107278	0.00243859	0.13323567
**mmu-miR-486a-3p**	-1.462469185	4.073335964	9.01261107	0.002681231	0.134284989
**mmu-miR-222-5p**	1.145689718	7.186149466	8.840555923	0.002946094	0.136200188
**mmu-let-7e-5p**	1.101592701	9.85892653	8.491639219	0.003567822	0.14794285
**mmu-miR-7009-5p**	2.748138071	0.968421332	8.390213736	0.003772465	0.14794285
**mmu-miR-125b-1-3p**	-1.319389637	6.230165876	8.311917735	0.003938578	0.14794285
**mmu-miR-214-5p**	-1.720090098	2.785304155	7.901662637	0.004938938	0.174605983
**mmu-miR-146b-3p**	1.283877215	3.432962824	7.740860324	0.005398507	0.180250166
**mmu-miR-451a**	-0.999415554	8.976131541	7.593093012	0.005859234	0.185336819
**mmu-miR-486b-5p**	-1.012507033	6.474143365	7.098884637	0.007713194	0.231781465

Supplementary Table 4. Comparison of top 20 significantly altered microRNAs in AcpM-treated BMDMs when compared to untreated cells. Log2 FC, log2 fold-change; Log2 CPM, log2 counts-per-million; LR, likelihood ratio statistics; P-Value, the two-sided p-value; FDR, false discovery rate.
